# The compensatory mechanism and clinical significance of hydrocephalus after cranioplasty

**DOI:** 10.3389/fneur.2022.1075137

**Published:** 2023-01-12

**Authors:** Xiansheng Qiu, Dong Wang, Li Chen, Guanlin Huang, Xiaoping Zhou, Qiang Chen, Zhanxiang Wang

**Affiliations:** ^1^The Graduate School of Fujian Medical University, Fuzhou, Fujian, China; ^2^Department of Neurosurgery, Ganzhou People's Hospital, Ganzhou, Jiangxi, China; ^3^Department of Neurosurgery, Fuzhou 900th Hospital of PLA, Fuzhou, Fujian, China

**Keywords:** hydrocephalus, ventriculoperitoneal shunt, decompressive, craniectomy, complications

## Abstract

**Objective:**

Cranioplasty (CP) and ventriculoperitoneal shunt (VPS) are procedures required after decompression of the flap (DC) to protect the cranial frame and prevent hydrocephalus. This study evaluated the safety and efficacy of different surgical sequences of CP and VPS after DC and identified risk factors for necessary permanent VPS.

**Methods:**

From January 2017 to December 2021, valid follow-up data were collected in 192 cases. The observation group preferred CP, and then evaluated whether to receive VPS according to the progress of hydrocephalus. the control group was prioritized for VPS and continued with CP after 1 week. The improvement of hydrocephalus symptoms, follow-up outcomes, and post-operative complications before and after surgery were compared between the two groups, and univariate analysis was used to determine the risk factors for necessary permanent risk factors for VPS.

**Results:**

There were 86 cases (44.8%) in the observation group, who received CP first, while 106 cases (55.2%) in the control group received VPS and CP, respectively. There was no significant difference between the two groups according to Barthel index, FMAS, Mrs, GCS, and Evans index, and there was no statistical difference in complications between the two groups. However, in the observation group, hydrocephalus disappeared after CP operation in 29 cases (33.7%), and finally avoided VPS. Univariate analysis showed that the main etiology was related to the size of the skull defect, the distance of the talus margin relative to the flap to the midline, and lumbar puncture pressure was a predictor of the need for permanent VPS.

**Conclusion:**

This study provides detailed information on the efficacy and complications of different sequences of preferential CP or VPS after DC surgery. We found that priority CP reduced the incidence of VPS surgery without affecting surgical outcomes and complications.

## 1. Introduction

Decompressive craniectomy (DC) is a life-saving surgery that is often used to treat traumatic brain injury (TBI), massive cerebral infarction(MCI), intracerebral hemorrhage (ICH) and subarachnoid hemorrhage (SAH) caused by a sharp increase in intracranial pressure (ICP) and cannot be controlled by drugs. The goal of DC is to preserve more neurological function by maintaining cerebral blood perfusion, ensuring oxygenation, and improving intracranial compliance (1, 2).

However, DC will result in many complications, among which hydrocephalus is the most common complication, the most common of which is hydrocephalus, with an incidence of up to 17.7% and a high mortality and disability rate (3–6). The main causes of hydrocephalus include the loss of bone flap support, the impact of atmospheric pressure outside the intracranial abnormal pressure, as well as local brain tissue in the bone window location and position shift, all of which result in varying degrees of cerebral perfusion disorders and cerebrospinal fluid circulation disorders, cerebrospinal fluid accumulation, and oppression subarachnoid and brain pool (7). Currently, cranioplasty (CP) and ventriculoperitoneal shunt are the two major treatments (VPS) (8) CP can effectively solve circulatory disorders and insufficient blood supply caused by skull defects and improve the appearance of patients, brain tissue safety and neurological function (9). VPS is the drainage of excess cerebrospinal fluid from the ventricles of the patient into the abdominal cavity and absorption, thereby improving the symptoms of hydrocephalus (10).

Current studies have shown that Simultaneous or staged CP and VPS have different advantages and disadvantages. However, there is still disagreement regarding whether to give CP or VPS precedence in staged surgery (11–13). However, clinical practice has proven that hydrocephalus disappears in some patients after CP, and VPS can be avoided in the later stage (14). Therefore, we retrospectively analyzed patients with CP and VPS after DC for hydrocephalus to find the population with the greatest benefit from free VPS after CP and clarify the compensation mechanism of hydrocephalus after CP.

## 2. Methods

### 2.1. Patients selection

From January 2017 to December 2021, we collected patients with hydrocephalus secondary to staged DC for CP and VPS. All patients were from the First Affiliated Hospital of Xiamen University, the People's Hospital of Ganzhou City, and the 900 Hospital of the PLA Joint Logistic Support Force. This study was approved by the institutional review Board of the hospital. Because of the retrospective nature of the study, the requirement for informed consent was waived.

Medical records were retrospectively reviewed for patient variables, including sex, age, primary causes (cerebral trauma, cerebral infarction, intracranial hemorrhage and subarachnoid hemorrhage, brain tumor, and others), skull defect parts and skull defect area, bone flap edge to the centerline distance, time of CP, underlying diseases (hypertension, diabetes), lumbar puncture pressure, intraoperative meningeal damage, and lumbar cisterna indwelling rate. Records of patients before and 6 months after the operation, Barthel Index, FMAS, Mrs, GCS, Evans' Index and perioperative complications (such as implant infection, subdural fluid, epilepsy, drainage disorder, bleeding).

Inclusion criteria: ① History of DC; ② hydrocephalus diagnosed by CT or MRI (15–17); (Evan's index >0.3, or associated with periventricular interstitial edema; Other causes of ventricular enlargement were excluded. ③ The presence of hydrocephalus-related symptoms, such as headache, neurological dysfunction, etc., normal pressure hydrocephalus contains at least one of the following symptoms: gait disorders, cognitive disorders, urinary incontinence (18–20). ④ At least 4 weeks after DC.

The exclusion criteria were as follows: ① CP and VPS were performed simultaneously; ② GCS score ≤5 and the preoperative cerebrospinal fluid release test was ineffective; ③ VPS and CP were prohibited, such as intracranial infection and abdominal infection; ④ pregnant women and children.

### 2.2. Patient management

**Figure 1:**
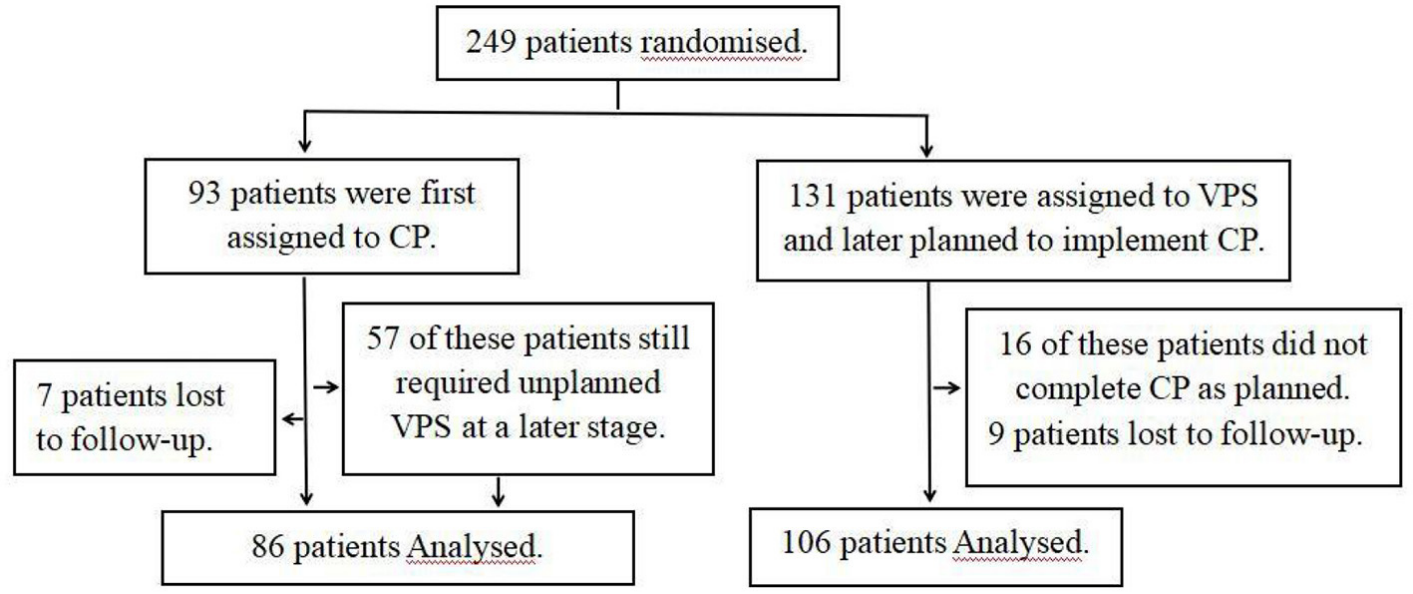
Study flowchart.

(1) Preoperative preparation
  A retrospective analysis was used in this study. All patients were divided into two groups according to the order of surgery, with the observation group being given priority for CP (including CP only and continued VPS without relief of hydrocephalus after CP) and the control group being given priority for VPS and continued CP after 1 week (Figure 1).

(2) Surgical methods
  Three lumbar punctures were conducted 7 days before to surgery to assess cerebrospinal fluid pressure and rule out intracranial infection. The average of the three punctures was used as the intracranial pressure value in the normal physiological condition with the skull intact. The lumbar puncture is performed in a lateral position with the skull defect side facing upward. A cerebrospinal fluid release test was also required for each lumbar puncture to exclude patients with negative cerebrospinal fluid release test. High-pressure hydrocephalus is defined as three consecutive lumbar punctures with an average pressure of more than 200 mmH_2_O. For these patients, we carry out continuous lumbar pool draining 24 h before to surgery, and lumbar pool drainage if there is a brain bulge in the bone window area until the bone window is flat. The syndrome of the trephined (SoT) and paradoxical herniation (PH) should be monitored during lumbar pool draining, and once it occurs, the drainage should be stopped and CP should be performed as soon as possible. Lumbar pool drainage is temporarily closed during surgery, It is recommended that the surgery be performed 3–6 months after DC surgery if the bone window dilates significantly after continuous lumbar cistern drainage and the skull model is difficult to place.

(3) Post-operative management
  All patients underwent CT examination 24 h after surgery to observe the progression of hydrocephalus and the detection of bleeding in the surgical area. The observation group also needed to remove the lumbar pool drainage tube as early as possible according to the patients' clinical manifestations and imaging data assessment. The cumulative duration of the lumbar pool did not exceed 10 days. If hydrocephalus is suspected of not resolving, another cerebrospinal fluid release test is required to determine the need for continued VPS after removal of the lumbar pool.

### 2.3. Follow-up

Patients were followed up post-operatively at 1, 3, 6, 12, and 18 months in outpatients, inpatients (including outpatients), and by telephone; the follow-up included the degree of improvement of neurological function and new symptoms and signs. The imaging results were determined by imaging and neurology physicians who reviewed the films together. Patients in the observation group should be followed up more frequently within 6 months and returned to the hospital for evaluation as soon as hydrocephalus symptoms or neurological decline is suspected.

### 2.4. Outcome assessment

The improvement effect of surgery was assessed based on the hydrocephalus symptoms (including cognitive living ability assessment, speech ability, physical activity ability, improvement of urinary incontinence, etc.,) and medical imaging (including reduction of ventricular morphology and remission of interstitial brain edema, etc. During the follow-up period. The incidence of post-operative complications was recorded for all patients and quantified using the Barthel Index (points), FMAS (points), Mrs (points), GCS (points) and Evan's index. A recurrence of hydrocephalus was defined as an Evan's index >0.3, which had to fall again after neurological improvement.

The primary endpoint event was defined as perioperative death from any cause. Reoperation to remove the skull titanium mesh or shunt was needed; any intracranial bleeding or severe infection required a second operation. The secondary endpoint events were a decrease in GOS of more than 1 point or a decrease in GCS of more than 2 points in coma patients; new-onset epilepsy, poor healing of the surgical mouth, subcutaneous effusion, cerebrospinal fluid leakage, etc.

## 3. Statistical analysis

Continuous variables are expressed as the mean ± standard deviation (range), and categorical data are expressed as frequencies and percentages. To assess risk factors for propensity to VPS and after CP surgery, one-way analysis was used, and a bilateral *p*-value < 0.05 was considered statistically significant. Data were analyzed using the Statistical Package for Social Sciences (SPSS, Version 22.0; IBM, Armonk, NY, USA).

## 4. Outcomes

### 4.1. Comparison of baseline characteristics between the observation and control groups

We received 249 patients who met the criteria for inclusion and exclusion, including 93 in the observation group, of whom 7 were lost to follow-up; and 131 were in the control group, of which 9 were lost to follow-up, and 16 refused to continue CP after bypass. Finally, we collected complete clinical data and follow-up information for 192 patients (observation group: 86; control group: 106) (Table 1).

**Table 1:** Comparison of baseline characteristics in patients with observation and control groups.

**Variable**	**Total (*n* = 192)**	**Observation group (*n* = 86)**	**Control group (*n* = 106)**	***p*-value**
Mean age (years)		61.3 ± 11.6	60.1 ± 13.2	0.509
Female	86	40 (46.0)	46 (43.4)	0.666
Initial presentation				0.211
TBI	45	18 (20.9)	27 (25.5)	
MCI	42	24 (27.9)	18 (17.0)	
ICH	46	24 (27.9)	22 (20.8)	
SAH	45	15 (17.4)	30 (28.3)	
Intracranial tumor	10	4 (4.7)	6 (5.7)	
Other	4	1 (1.2)	3 (2.8)	
Cranial defect site				0.099
Left	81	33 (38.4)	48 (45.3)	
Right	83	44 (51.2)	39 (36.8)	
Bilateral	28	9 (10.5)	19 (17.9)	
Cranial defect size				0.604
≤100 mm	92	43 (50.0)	49 (46.2)	
>100 mm	100	43 (50.0)	57 (53.8)	
Midline distance				0.646
Midline distance ≤25 mm	104	45 (52.3)	59 (55.7)	
Midline distance >25 mm	88	41 (47.7)	47 (44.3)	
Cranioplasty time				0.009
<3 months	74	42 (48.8)	32 (30.2)	
≥3 months	118	44 (51.2)	74 (69.8)	
Basic illness				
Hypertension	43	16 (18.6)	27 (25.5)	0.256
Diabetes	34	13 (15.1)	21 (19.8)	0.397
Intracranial pressure				0.454
High -pressure	91	39 (45.3)	52 (49.1)	
sNPH	83	41 (47.7)	42 (39.6)	
Low-pressure	18	6 (7.0)	12 (11.3)	
Intraoperative dural damage	22	9 (10.5)	13 (12.3)	0.697
lumbar cistern drainage	68	31 (36.0)	37 (34.9)	0.869

TBI, traumatic brain injury; MCI, massive cerebral infarction; SAH, subarachnoid hemorrhage; ICH, intracerebral hemorrhage; sNPH, secondary normal pressure hydrocephalus.

### 4.2. Comparison of surgical effects between the observation and control groups

There was no significant difference in the Barthel Index (points), FMAS (points), Mrs score, GCS (points) and Evan, sor Evan's index between the two groups before surgery (*P* > 0.05), 6 months later, the Barthel Index (points), FMAS (points), Mrs score and GCS (points) levels in both groups were significantly increased, while Evan's index was significantly decreased. The difference was significant before and after surgery (*P* < 0.05), but there was no significant difference between the observation group and the control group at the same stage (Table 2).

**Table 2:** Comparison of neurological function scores and hydrocephalus index before and after surgery in the observation and control groups.

**GROUP (*n* = 192)**	**Barthel index**		**FMAS**		**mRs**		**GCS**		**Evans' index**	
	**PRE-OP**	**POST-30d**	**PRE-OP**	**POST-30d**	**PRE-OP**	**POST-30d**	**PRE-OP**	**POST-30d**	**PRE-OP**	**POST-30d**
Observation group (*n* = 86)	63.37 ± 14.92	79.83 ± 19.14	55.1 ± 14.01	91.85 ± 26.99	3.08 ± 0.92	1.9 ± 1.39	10.36 ± 2.53	12.76 ± 2.51	0.37 ± 0.04	0.23 ± 0.02
Control group (*n* = 106)	62.83 ± 18.25	78.02 ± 21.99	54.08 ± 18.55	91 ± 26.7	3.32 ± 0.97	1.83 ± 1.36	10.26 ± 2.48	12.79 ± 2.06	0.37 ± 0.05	0.23 ± 0.03
t/χ^2^	0.222	0.6	0.425	0.218	−1.736	0.328	0.265	−0.111	−0.156	0.324
*P*	0.825	0.549	0.671	0.828	0.084	0.744	0.791	0.912	0.876	0.747

Barthel Index, the Barthelindex of ADL; FMAS, Fugl-Meyer assessment scale; mRs, Modified Rankin Scale; GCS, Glasgow coma scale.

Evans' index: the ratio between the maximum width of the frontal horns and the maximal internaldiameter of the skull at the level of Monro's foramens. A radiological diagnosis of hydrocephalus is defined as an Evans' index value > 0.30.PRE-OP, pre-operation. POST-30d, post-operation 30 days.

### 4.3. Comparison of surgical complications between the observation and control groups

After excluding patients who had epilepsy before surgery but still had epilepsy or aggravated epilepsy after surgery. There was no significant difference in the prognosis of disability or complication rate between the two groups (*P* > 0.05). In the observation group, one patient had an intracranial infection with an infection rate of 1.1% (1/86), which was cured after anti-infection treatment; two cases had subdural effusion, which was treated with intensive rehydration and hyperbaric oxygen for improvement, and two cases had epilepsy, which was considered to be caused by thermal stimulation of the surgical electric knife and was treated with antiepilepsy medication, and 6 months after quitting the medication, no recurrence was noted. In the control group, two cases of intraoperative infection, three cases of subdural fluid and four cases of epilepsy disappeared after drug treatment; one case of new intracranial hemorrhage of ~5 ml was seen on post-operative review, which was considered to be caused by intraoperative ventricular puncture during VPS, with no new neurological dysfunction after conservative treatment and rehabilitation (Table 3).

**Table 3:** Comparison of complications between the observation and control groups.

**Complication**	**CP (86)**	**VPS (106)**	***P*-value**
Total (%)	6 (7.0)	13 (12.3)	*P >* 0.05
Implant infection	1 (1.2)	2 (1.9)	1
Subdural fluid	2 (2.3)	3 (3.8)	1
Epilepsy	2 (2.3)	4 (3.8)	0.7
Drainage disorder	-	1 (0.9)	1
Bleeding	-	3 (2.8)	0.25

Values are presented as numbers (%).

The complications in this study were slightly lower than those in other studies. Except for the fact that the existing complications were not included, the reason may be that 29 patients in the observation group only received CP but not VPS, which reduced the number of operations. Secondly, the complications of staged operations were less than those of simultaneous operations (11).

### 4.4. Comparison of the follow-up period between the observation and control groups

In the observation group, 57 patients with unremitting hydrocephalus were given continued VPS during the follow-up period. Fifteen (26.3%) of the 57 patients in this group had TBI as the primary cause, 13 (22.8%) had SAH, 34 (59.6%) had bone flaps >100 mm, 41 (71.9%) had non-isobaric hydrocephalus, and 39 (68.4%) were <25 mm from the midline. In the control group, one patient had poor shunt drainage, 3 months later, the shunt adapter was found to be dislodged, and the shunt valve was replaced and continued to be fixed. One patient had recurrent fever 5 months after surgery, and intracranial infection was diagnosed by lumbar puncture, which was ineffective after conservative treatment and was diagnosed as calculous cholecystitis and retrograde infection to the skull and was cured after removal of the gallbladder and continued anti-infection treatment.

### 4.5. Analysis of risk factors for non-planed VPS after CP

In the observation group, 29 of the 86 patients with hydrocephalus gradually resolved after CP and did not undergo VPS again during the follow-up period. The following variables were used to assess risk factors for the need for VPS after CP: gender, age, primary cause (TBI, MCI, ICH and SHA, brain tumor, and others), skull defect parts and skull defect area, bone flap edge to the centerline distance, time of CP, lumbar puncture pressure; and by univariate logistic regression analysis, primary etiology, skull defect area, distance from the edge of the bone flap to the midline, and lumbar puncture pressure were the risk factors for assessing the need for VPS (Table 4).

**Table 4:** Risk factor analysis for non-planed VPS after CP.

**Variables**	**Non-planed VPS**		**Univariate analysis**	
	**(+) *n* = 29**	**(–) *n* = 57**	**OR (95% CI)**	***p*-value**
Male	15	31	1.11 (0.45–2.73)	0.815
Age (≥60 years)	15	30	1.04 (0.42–2.54)	0.937
TBI	3	15	4 (1.07–14.96)	0.039
Cerebral infarction	10	14	0.62 (0.23–1.64)	0.334
ICH	9	15	0.79 (0.3–2.12)	0.645
SAH	2	13	4.2 (1.02–22.78)	0.047
Intracranial tumor	4	0	2.66 (0.80–8.80)	0.110
Unilateral Cranial defect	26	51	0.98 (0.23–4.24)	0.979
PC interval after DC > 3 M	18	26	0.51 (0.21–1.28)	0.152
Non-NPH	4	41	16.02 (4.81–53.35)	<0.01
Cranial defect size	9	34	3.29 (1.27–8.48)	0.014
Midline distance ≤25 mm	6	39	8.31 (2.88–23.92)	<0.01

Values are presented as numbers (%).

Non-planed VPS, non-planed ventriculoperitoneal shunt; OR, odds ratio; CI, confidence interval; TBI, traumatic brain injury; SAH, subarachnoid hemorrhage; ICH, intracerebral hemorrhage; Non-NPH, non-normal pressure hydrocephalus. Normal pressure is defined as 70–200 mmH_2_O.

## 5. Discussion

### 5.1. The compensatory effect of hydrocephalus after CP has not attracted enough cognition or attention

DC is a common emergency surgery to suppress malignant intracranial hypertension, which has irreplaceable advantages in reducing the mortality and disability rate. However, 17.7% of patients will still develop hydrocephalus (3, 5, 13, 21, 22). Although VPS can restore normal cerebral fluid circulation and relieve hydrocephalus symptoms, additional VPS not only increases the burden and pain of patients but also increases the risk of infection, bleeding, epilepsy, poor skin healing, implant exposure, and rejection (23, 24). Various protocols have been tried to reduce complication rates (25). For example, studies have suggested simultaneous VPS and CP to improved clinical outcomes, shorter hospital stays, and lower medical costs (26). However, some researchers believe that staged surgery can reduce the single operation time, thereby reducing the risk of surgical complications such as infection (11, 13).

There is still no consensus on whether VPS or CP should be prioritized in staged surgery for the population that will benefit the most, but in clinical practice, some patients with subsequent disappearance of hydrocephalus after CP are eventually spared from VPS (14, 27). In this study, 29 out of 86 patients in the observation group did not need VPS after priority CP, which also confirmed the above view again, which provides important reference significance for us to choose the surgical sequence. Through univariate logistic regression analysis of the data of 29 patients who did not need VPS and the remaining 57 patients who underwent VPS again, we found that the primary etiology, bone flap size, bone flap distance from the midline and intracranial pressure were risk factors for the evaluation of the need for VPS, which may be related to the different compensatory abilities of hydrocephalus after CP.

### 5.2. The influence of the skull on cerebrospinal fluid dynamics

Because DC alters the sealing and integrity of the skull cavity, intracranial pressure, cerebral hemodynamics, and cerebrospinal fluid dynamics will be affected, and these changes can be evaluated by cerebral compliance (ΔV/ΔP). All factors that cause the increase in cerebral compliance will reduce the pulsation of cerebrospinal fluid, causing cerebrospinal fluid dynamics to be disrupted (28–31). Grant made the first report on the complication SoT after DC (32). Although the pathophysiological mechanisms of this pathology have not been fully elucidated, the influences proposed by various theories may end up being related to cerebrospinal fluid dynamics (33). Since the volume in the cranial cavity is relatively fixed, the blood, cerebrospinal fluid, and brain tissue homeostasis are in a reciprocal relationship. SoT symptoms can disappear after CP, which fully suggests that SoT is a reversible complication after DC. This type of hydrocephalus is not permanent, and its pathological changes may be a transient disturbance of cerebrospinal fluid dynamics after DC, and with the recovery of cerebrospinal fluid dynamics after CP (34), cerebral blood flow increases and brain tissue pressure is relieved, they can return to normal physiology again (35).

Secondly, the larger the bone flap removed, the greater the scalp malleability, the higher the brain compliance, and the greater the risk of cerebrospinal fluid dynamics disorder. Some studies have shown that even without external drainage of cerebrospinal fluid or massive dehydration with drugs, patients with large bone flap decompression still have flap depression and abnormal cerebral hernia. Therefore, a large bone flap may have a significant impact on cerebrospinal fluid dynamics (36, 37). Some retrospective studies have found that removing bone flaps larger than 70 mm increases the risk of hydrocephalus (38, 39). The subdural effusion in the oversized bone flap group was significantly higher than that in the conventional bone flap group, which also suggested the influence of the size of the removed bone flap on the abnormal distribution of cerebrospinal fluid (40).

As a result, cerebrospinal fluid dynamics disorder after DC results in abnormal distribution of cerebrospinal fluid. If the influence of cerebrospinal fluid dynamics after DC is to be reduced, it is necessary to priority its return to a normal physiological state and restore the integrity of the skull as soon as possible. With the improvement and recovery of cerebrospinal fluid dynamics after DC, hydrocephalus itself is less affected in patients with small and medium DC, and the probability of cerebrospinal fluid dynamics returning to normal after DC is greater than that in patients with large DC.

### 5.3. Increased absorption of cerebral effusion by arachnoid granules

The central nervous system is physically protected by Cerebrospinal fluid (CSF), which also supplies the homeostatic environment required for healthy neuronal function (31). During the production and absorption of cerebrospinal fluid, it is not stationary but continues to beat and remains relatively stable. Cerebrospinal fluid is secreted by choroid plexus epithelial cells, flows along specific pathways and is finally absorbed by venous sinuses. Any cause that affects increased secretion of cerebrospinal fluid, or (and) absorption disorders, or (and) circulation disorders can disrupt the homeostasis of cerebrospinal fluid circulation (41, 42). For example, SHA, intraventricular hemorrhage, or intracranial infection are more likely to develop into hydrocephalus due to the release of hemoglobin and transforming growth factor-β1, which mechanically block the absorption of cerebrospinal fluid and are more difficult to recover from than those with less mechanical obstruction (43, 44).

According to earlier research by Waziri, hydrocephalus still manifests after DC in cases of ischemic stroke even though subarachnoid hemorrhage does not occlude the arachnoid granules. Consequently, it was believed that the primary factor causing hydrocephalus following DC was a blockage of cerebrospinal fluid absorption and output (14). Because the nutricular granule is a pressure-dependent unidirectional valve structure, the absorption of cerebrospinal fluid needs to be achieved under a specific pressure (45). Therefore, once the arachnoid granules obstruct the outflow of CSF and the absorption of hydrocephalus, the risk of hydrocephalus absorption is greatly increased. It has also been demonstrated that the closer the bone flap is to midline of the skull, the greater the probability of hydrocephalus, which may be related to the greater distribution of arachnoid granules near the midline of the skull (3, 46). As a result of the pressure recovery, arachnoid granules increase the absorption of cerebrospinal fluid, reducing the degree of hydrocephalus (47, 48). In brief, patients with bone flaps farther from the midline benefited more than those with bone flaps <25 mm.

### 5.4. Effects of intracranial and extracranial pressure balance and improved cerebral blood flow

The normal circulation of cerebrospinal fluid depends on stable intracranial pressure (ICP), which is maintained by arterial pulsation to provide cerebrospinal fluid flow power (49). Fodstad proposed that the pathogenesis of SoT may be the direct effect of atmospheric pressure on the cerebral cortex and intracranial venous return due to the cranial defect, resulting in a siphoning effect on cerebrospinal fluid dynamics, leading to a negative gradient between atmospheric pressure and intracranial pressure (50). In summary, after DC, scalp ductility increased, the intracranial pressure environment was broken, ICP decreased, and the speed of cerebrospinal fluid circulation slowed. The study of cerebrospinal fluid dynamics by magnetic resonance technology also confirmed that the phenomenon of cerebrospinal fluid flow velocity slowed down after DC (50). Therefore, cerebrospinal fluid accumulates continuously in the ventricle, resulting in higher and higher pressure to increase pulsing pressure to promote cerebrospinal fluid flow. After DC, due to a “squeeze pressure” on the ventricle space, it is no longer necessary to rely on passive ventricular expansion to provide pressure, and the cerebrospinal fluid flow rate can be increased by two times (51), which allows part of the excess cerebrospinal fluid to be expelled and absorbed. Stula also reported that cranial repair ameliorated cortical compression (52). Thus, restoration of physiological structure of CP can break the disorder of cerebrospinal fluid circulation after DC, reduce the probability of hydrocephalus and improve neurological function.

In addition to the effect of intracranial pressure on cerebrospinal fluid circulation, the study also found that cerebral blood flow velocity decreased after bone flap removal, and the larger the bone flap was, the slower the blood flow velocity (53, 54). The Richaud study also concluded that CP could increase cortical blood flow on the side of the skull defect by 15–30% (55, 56). Second, Yamaura found that 95% of patients with DC had accompanying EEG changes, and 60% of these patients had EEG improvements after CP, as well as clinical seizures (57). In addition, with the increase in rehabilitation programs, brain function training and recovery after CP, some patients can relieve the degree of hydrocephalus through compensation of the body.

## 6. Limitations

The present study was a small sample size, with only 29 patients exempted from VPS in the preliminary results and lack of clinical data in children, and the final conclusion remains to be confirmed; therefore, patients without VPS for CP alone need to be followed up more intensively with timely review and evaluation for VPS at any time to prevent neurological damage from hydrocephalus. Since neurotrauma service can positively impact the management of the broad spectrum of TBI in different clinical settings (58); subsequently we will seek patients' consent to share the follow-up information to the neurotrauma unit specialists in the neurotrauma unit and the neurorehabilitation unit to standardize patient management and enable patients to receive more standardized care.

## 7. Conclusion

DC is prone to pathological changes such as disturbed cerebrospinal fluid dynamics, impaired hydrocephalus absorption, changes in intracranial and extracranial pressure, and decreased cerebral blood flow. Priority CP can assist hydrocephalus patients in returning to normal physiology as quickly as possible without impairing cerebral function, and there is a potential to be free of VPS. Additionally, the primary etiology, the location of the skull defect, the distance from the bone flap's border to the midline, and the pressure of the lumbar puncture are crucial in determining whether or not a permanent VPS is necessary.

## Data availability statement

The original contributions presented in the study are included in the article/supplementary material, further inquiries can be directed to the corresponding author.

## Ethics statement

The studies involving human participants were reviewed and approved by Ethics Committee of Ganzhou People's Hospital. The patients/participants provided their written informed consent to participate in this study.

## Author contributions

DW: study design and collection, analysis, and interpretation of data. XQ: data collection, manuscript preparation, and review. GH and QC: analysis of images. LC and XZ: data collection. ZW: study concept and design, analysis of images, and critical revision of the manuscript for intellectual content. All authors contributed to the article and approved the submitted version.
